# Checklist of the Cecidomyiidae (Diptera) of Finland

**DOI:** 10.3897/zookeys.441.7503

**Published:** 2014-09-19

**Authors:** Mathias Jaschhof, Marcela Skuhravá, Jouni Penttinen

**Affiliations:** 1 Thälmann-Ring 64, D-17491 Greifswald, Germany; 2 Bítovská 1227/9, 140 00 Praha 4, Czech Republic; 3 Metsähallitus, Natural Heritage, P.O. Box 36, FIN-40101 Jyväskylä, Finland

**Keywords:** Gall midges, Finland, species list

## Abstract

A list of the 356 species of Cecidomyiidae (Diptera) recorded from Finland is presented, which comprises 6 Lestremiinae, 156 Micromyinae, 16 Winnertziinae, 69 Porricondylinae, and 109 Cecidomyiinae. The faunistic knowledge of Finnish Winnertziinae, Porricondylinae and Cecidomyiinae is regarded as particularly poor. Based on species numbers known from other countries in Europe, a conservative estimate is 700–800 species of Cecidomyiidae actually occurring in Finland.

## Introduction

With a world total of more than 6200 species described, Cecidomyiidae are among the largest families of Diptera, although adult cecidomyiids are mostly delicate midges with body lengths of less than 5 mm. The family has been catalogued (Gagné and Jaschhof 2014) and that publication provides bibliographic and other information on all known species. Six subfamilies are recognised: the fungivorous Catotrichinae, Lestremiinae, Micromyinae, Winnertziinae and Porricondylinae, and the mostly herbivorous Cecidomyiinae. The latter subfamily, which also includes fungivores and predators, comprises the largest gall-making group of insects (hence the family name that translates as gall midges).

In the fungivorous subfamilies, many larvae are associated with dead wood, rendering those a biodiverse, albeit little studied group of saproxylic insects. In Cecidomyiinae, larvae associated with living flowering plants are usually restricted to development on one, or a few closely-related host species; predators are also generally assumed to be highly host-specific, as far as is known.

The Palaearctic has more named Cecidomyiidae species than any other zoogeographical region, and the best known gall midge fauna is that of Europe, with more than 1800 species (Skuhravá 2006). Even so, European Cecidomyiidae are far from being completely known, and the Finnish fauna provides a good example. To illustrate, while the previous checklist of Finnish Cecidomyiidae (Hackman 1980) comprised only four species of Lestremiinae and Micromyinae, a recent taxonomic study increased that number to 162, including 34 new species (Jaschhof and Jaschhof 2009). As for Winnertziinae and Porricondylinae, 27 species in Hackman’s (1980) list compare with 69 species recorded here (Penttinen and Siitonen 2005, Penttinen and Spungis 2007, Penttinen and Jaschhof 2009; Jaschhof, unpublished; Penttinen, unpublished), yet much remains to be discovered, as is testified by specimens in the Penttinen Collection belonging to a number of unnamed species. In Cecidomyiinae, only three species (Hulden 2003; Skuhravá, unpublished) are here added to the Hackman list, a meager increase that is obviously explained by the slight research effort in recent years.

Identification of cecidomyiids, both larvae and adults, is generally difficult and usually left to a few taxonomic specialists of the family. An adult key to the Palaearctic genera of Cecidomyiidae was provided by Skuhravá (1997); to the Holarctic genera of Lestremiinae and Micromyinae by Jaschhof and Jaschhof (2009), and to the Holarctic genera of Winnertziinae and Porricondylinae by Jaschhof and Jaschhof (2013). Adult keys and illustrations by Jaschhof and Jaschhof (2009, 2013) can be used to identify the Finnish species of the fungivorous subfamilies. Adult keys to identify species of Cecidomyiinae in Finland are only available for a few small groups, so any attempts in this regard rely largely on descriptions and illustrations scattered in an extensive literature. In general, many Cecidomyiinae species cannot be identified unless a bundle of information (regarding host, gall, immature stages, adults of both sexes) are known. Illustrated keys to galls, including those caused by gall midges, have been published for Central and Northern Europe by Buhr (1964, 1965), a resource that is currently being revised and updated for publication in English in 2015 (see http://www.pflanzengallen.de).

The arrangement of taxa in this list largely follows the recent edition of the world catalogue (Gagné and Jaschhof 2014) except that genera are here arranged under their pertinent tribes and supertribes, if known. All Finnish records of species of the fungivorous subfamilies were revised in recent years (Jaschhof and Jaschhof 2009, 2013; Penttinen, unpublished), so can be related to actual voucher specimens deposited in museum collections. The list of Cecidomyiinae, which is not updated to the same extent, is compiled from published locality records based on varying evidence, including galls alone.

**Number of species:**

World: 6203 species (Gagné and Jaschhof 2014)

Europe: 1800 species (Skuhravá 2006)

Finland: 356 species (this paper)

Faunistic knowledge level in Finland: poor

## Checklist

Nematocera Dumeril, 1805

infraorder Bibionomorpha Hennig, 1954

superfamily Sciaroidea Billberg, 1820

**CECIDOMYIIDAE** Newman, 1834

LESTREMIINAE Rondani, 1840

tribe Lestremiini Rondani, 1840

***ALLARETE*** Pritchard, 1951

*Allarete nigra* Mamaev, 1994

***ANARETE*** Haliday, 1833

*Anarete* sp. 2 [see Note below]

***ANARETELLA*** Enderlein, 1911

*Anaretella defecta* (Winnertz, 1870)

= *magnicornis* Mamaev, 1964

*Anaretella iola* Pritchard, 1951

***LESTREMIA*** Macquart, 1826

*Lestremia cinerea* Macquart, 1826

*Lestremia leucophaea* (Meigen, 1818)

MICROMYINAE Rondani, 1856

tribe Acoenoniini Pritchard, 1960

***ACOENONIA*** Pritchard, 1947

*Acoenonia europaea* Mamaev, 1964

tribe Aprionini Jaschhof, 1998

***APRIONUS*** Kieffer, 1894

*Aprionus accipitris* Jaschhof, 1997

*Aprionus acutus* Edwards, 1938

*Aprionus adventitius* Jaschhof, 2009

*Aprionus aquilonius* Jaschhof, 2009

*Aprionus arcticus* Mamaev, 2001

*Aprionus aviarius* Mamaev & Berest, 1990

*Aprionus betulae* Jaschhof, 1996

*Aprionus bidentatus* (Kieffer, 1894)

*Aprionus bifidus* Mamaev, 1963

*Aprionus bispinosus* Edwards, 1938

*Aprionus brachypterus* Edwards, 1938

*Aprionus brevitegminis* Jaschhof, 2009

*Aprionus cardiophorus* Mamaev, 1963

*Aprionus carinatus* Jaschhof, 1996

*Aprionus complicatus* Mamaev & Berest, 1986

*Aprionus confusus* Mamaev, 1969

*Aprionus corniculatus* Mamaev, 1963

*Aprionus cornutus* Berest, 1986

*Aprionus dalarnensis* Mamaev, 1998

*Aprionus dentifer* Mamaev, 1965

*Aprionus dispar* Mamaev, 1963

*Aprionus dissectus* Mamaev & Berest, 1990

*Aprionus duplicatus* Mamaev, 1998

*Aprionus ensiferus* Jaschhof, 1996

*Aprionus fennicus* Jaschhof, 2009

*Aprionus flavidus* (Winnertz, 1870)

*Aprionus foliosus* Jaschhof, 2009

*Aprionus gladiator* Jaschhof, 2009

*Aprionus halteratus* (Zetterstedt, 1852)

*Aprionus heothinos* Jaschhof, 2009

*Aprionus hintelmannorum* Jaschhof, 2009

*Aprionus inquisitor* Mamaev, 1963

*Aprionus insignis* Mamaev, 1963

*Aprionus laevis* Mohrig, 1967

*Aprionus lapponicus* Jaschhof & Mamaev, 1997

*Aprionus laricis* Mamaev & Jaschhof, 1997

*Aprionus latitegminis* Jaschhof, 2009

*Aprionus longicollis* Mamaev, 1963

*Aprionus longitegminis* Yukawa, 1967

*Aprionus marginatus* Mamaev, 1963

*Aprionus miki* Kieffer, 1895

*Aprionus oligodactylus* Jaschhof, 2009

*Aprionus piceae* Jaschhof, 1997

*Aprionus praecipuus* Jaschhof, 2009

*Aprionus pyxidiifer* Mamaev, 1998

*Aprionus reduncus* Jaschhof, 2009

*Aprionus separatus* Mamaev & Jaschhof, 1997

*Aprionus sievertorum* Jaschhof, 2009

*Aprionus similis* Mamaev, 1963

*Aprionus spiniferus* Mamaev & Berest, 1990

*Aprionus spiniger* (Kieffer, 1894)

*Aprionus stiktos* Jaschhof, 2009

*Aprionus stylifer* Mamaev, 1998

*Aprionus styloideus* Mamaev & Berest, 1990

*Aprionus subacutus* Jaschhof, 1997

*Aprionus svecicus* Jaschhof, 1996

*Aprionus taigaensis* Jaschhof, 2009

*Aprionus tiliamcorticis* Mamaev, 1963

*Aprionus victoriae* Jaschhof, 2009

tribe Bryomyiini Berest, 1993

***BRYOMYIA*** Kieffer, 1895

= *Tomonomyia* Berest, 1993

*Bryomyia apsectra* Edwards, 1938

*Bryomyia bergrothi* Kieffer, 1895

*Bryomyia gibbosa* (Felt, 1907)

*Bryomyia producta* (Felt, 1908)

***HETEROGENELLA*** Mamaev, 1963

= *Cervuatina* Berest, 1993

*Heterogenella finitima* Mamaev, 1998

*Heterogenella hybrida* Mamaev, 1963

*Heterogenella linearis* Yukawa, 1971

***SKUHRAVIANA*** Mamaev, 1963

*Skuhraviana triangulifera* Mamaev, 1963

tribe Campylomyzini Kieffer, 1898

***CAMPYLOMYZA*** Meigen, 1818

*Campylomyza abbreviata* Jaschhof, 2009

*Campylomyza aemula* Mamaev, 1998

*Campylomyza alpina* Siebke, 1863

*Campylomyza arcuata* Jaschhof, 2009

*Campylomyza armata* Mamaev, 1963

*Campylomyza cavitata* Mamaev, 1998

*Campylomyza cingulata* Jaschhof, 2009

*Campylomyza dilatata* Felt, 1907

*Campylomyza falcifera* Jaschhof, 2009

*Campylomyza flavipes* Meigen, 1818

= *pallipes* Zetterstedt, 1850

*Campylomyza furva* Edwards, 1938

*Campylomyza fusca* Winnertz, 1870

*Campylomyza inornata* Jaschhof, 2009

*Campylomyza insolita* Jaschhof, 2009

*Campylomyza ormerodi* (Kieffer, 1913)

*Campylomyza serrata* Jaschhof, 1998

*Campylomyza spatulata* Mamaev, 1998

*Campylomyza stegetfore* Jaschhof, 2009

***EXCRESCENTIA*** Mamaev & Berest, 1991

*Excrescentia mutuata* Mamaev & Berest, 1991

***NEUROLYGA*** Rondani, 1840

= *Cordylomyia* Felt, 1911

*Neurolyga acuminata* Jaschhof, 2009

*Neurolyga bilobata* (Mamaev & Rozhnova, 1982)

*Neurolyga excavata* (Yukawa, 1967)

*Neurolyga interrupta* Jaschhof, 2009

*Neurolyga lonsdalensis* Jaschhof, 2009

*Neurolyga paludosa* Jaschhof, 2009

*Neurolyga sylvestris* (Felt, 1907)

*Neurolyga verna* (Mamaev, 1963)

tribe Catochini Edwards, 1938

***CATOCHA*** Haliday, 1833

*Catocha incisa* Jaschhof, 2009

*Catocha latipes* Haliday, 1833

tribe Micromyini Rondani, 1856

***MICROMYA*** Rondani, 1840

*Micromya lucorum* Rondani, 1840

***MONARDIA*** Kieffer, 1895

= *Pezomyia* Kieffer, 1913

**sg. *Monardia*** Kieffer, 1895

*Monardia abnormis* Mamaev, 1963

*Monardia armata* Jaschhof, 2003

*Monardia lignivora* (Felt, 1907)

*Monardia obsoleta* Edwards, 1938

*Monardia pediculata* (Mamaev, 1993)

*Monardia stirpium* Kieffer, 1895

*Monardia yasumatsui* Yukawa, 1967

**sg. *Trichopteromyia*** Williston, 1896

*Monardia magnifica* Mamaev, 1963

*Monardia relicta* Jaschhof, 2009

**sg. *Xylopriona*** Kieffer, 1913

*Monardia adentis* Jaschhof, 1998

*Monardia atra* (Meigen, 1804)

*Monardia monotheca* Edwards, 1938

*Monardia radiella* Mamaev, 1993

*Monardia toxicodendri* (Felt, 1907)

*Monardia unguifera* Berest & Mamaev, 1997

***POLYARDIS*** Pritchard, 1947

*Polyardis adela* Pritchard, 1947

*Polyardis bispinosa* (Mamaev, 1963)

*Polyardis silvalis* (Rondani, 1840)

tribe Peromyiini Kleesattel, 1979

***PEROMYIA*** Kieffer, 1894

*Peromyia abdita* Jaschhof, 2009

*Peromyia abnormis* Mamaev & Berest, 1990

*Peromyia albicornis* (Meigen, 1830)

*Peromyia anatina* Mamaev & Berest, 1990

*Peromyia angellifera* Jaschhof, 1997

*Peromyia anisotoma* Mamaev, 1994

*Peromyia apposita* Jaschhof, 1997

*Peromyia bicolor* (Edwards, 1938)

*Peromyia bidentata* Berest, 1998

*Peromyia boreophila* Jaschhof, 2001

*Peromyia caricis* (Kieffer, 1901)

*Peromyia concitata* Mamaev & Berest, 1994

*Peromyia cornuta* (Edwards, 1938)

*Peromyia curta* Jaschhof, 1997

*Peromyia diadema* Mamaev, 1963

*Peromyia edwardsi* Berest, 1994

*Peromyia fagiphila* Jaschhof, 1997

*Peromyia fungicola* (Kieffer, 1901)

*Peromyia intermedia* (Kieffer, 1895)

*Peromyia mitrata* Jaschhof, 1997

*Peromyia modesta* (Felt, 1907)

*Peromyia monilis* Mamaev, 1965

*Peromyia muscorum* (Kieffer, 1895)

*Peromyia ovalis* (Edwards, 1938)

*Peromyia palustris* (Kieffer, 1895)

*Peromyia perpusilla* (Winnertz, 1870)

*Peromyia photophila* (Felt, 1907)

*Peromyia pumila* Jaschhof, 2001

*Peromyia ramosa* (Edwards, 1938)

*Peromyia ramosoides* Jaschhof, 2009

*Peromyia sanguinea* (Kieffer, 1894)

*Peromyia scirrhosa* Jaschhof, 2009

*Peromyia scutellata* Mamaev, 1990

*Peromyia semotoides* Jaschhof, 2009

*Peromyia subbicolor* Jaschhof, 2009

*Peromyia subborealis* Jaschhof, 1997

*Peromyia syltefjordensis* Jaschhof, 1996

*Peromyia truncata* Yukawa, 1967

*Peromyia tundrae* Jaschhof, 1996

*Peromyia upupoides* Jaschhof, 1997

Winnertziinae Panelius, 1965

tribe Diallactiini Jaschhof, 2013

***Diallactia*** Gagné, 2004

= *Diallactes* Kieffer, 1894 preocc.

*Diallactia crocea* (Kieffer, 1894)

= *obscuripes* (Spungis, 1985)

***Sylvenomyia*** Mamaev & Zaitzev, 1998

*Sylvenomyia fennica* Penttinen & Jaschhof, 2009

*Sylvenomyia spinigera* (Spungis, 1985)

tribe Heteropezini Schiner, 1868

***Heteropeza*** Winnertz, 1846

*Heteropeza pygmaea* Winnertz, 1846

***Leptosyna*** Kieffer, 1894

*Leptosyna similis* Jaschhof, 2013

***Miastor*** Meinert, 1864

*Miastor metraloas* Meinert, 1864

tribe Winnertziini Panelius, 1965

***KRONOMYIA*** Felt, 1911

*Kronomyia ovalis* (Mamaev, 1964)

***RHIPIDOXYLOMYIA*** Mamaev, 1964

*Rhipidoxylomyia brevicornis* Mamaev, 1964

***Winnertzia*** Rondani, 1860

*Winnertzia bulbifera* Mamaev, 1963

*Winnertzia curvata* Panelius, 1965

*Winnertzia fusca* Kieffer, 1901

*Winnertzia globifera* Mamaev, 1963

*Winnertzia graduata* Spungis, 1992

*Winnertzia nigripennis* Kieffer, 1896

*Winnertzia rotundata* Spungis, 1992

*Winnertzia solidaginis* Felt, 1907

= *calciequina* Felt, 1907

Porricondylinae Kieffer, 1913

tribe Asynaptini Rübsaamen & Hedicke, 1926

***Asynapta*** Loew, 1850

*Asynapta breviata* Spungis, 1988

*Asynapta inflata* Spungis, 1988

*Asynapta magdalini* Panelius, 1965

*Asynapta pectoralis* (Winnertz, 1853)

*Asynapta rufomaculata* Panelius, 1965

*Asynapta saliciperda* Felt, 1908

= *populnea* Panelius, 1965

*Asynapta strobi* (Kieffer, 1920)

***Camptomyia*** Kieffer, 1894

*Camptomyia abnormis* Mamaev, 1961

*Camptomyia calcarata* Mamaev, 1964

*Camptomyia corticalis* (Loew, 1851)

*Camptomyia flavocinerea* Panelius, 1965

*Camptomyia gigantea* Spungis, 1989

*Camptomyia pinicola* Mamaev, 1961

*Camptomyia piptopori* Panelius, 1965

*Camptomyia regia* Spungis, 1989

*Camptomyia salicicola* Mamaev, 1961

= *populicola* Mamaev, 1961

*Camptomyia spinifera* Mamaev, 1961

***COLOMYIA*** Kieffer, 1892

*Colomyia clavata* Kieffer, 1892

***Parasynapta*** Panelius, 1965

*Parasynapta intermedia* Panelius, 1965

tribe Dicerurini Mamaev, 1966

= Solntseviini Mamaev, 1966

***Dicerura*** Kieffer, 1898

*Dicerura furculata* Mamaev, 1968

*Dicerura mixta* Spungis, 1987

*Dicerura rossica* (Mamaev, 1960)

*Dicerura triangularis* Mamaev, 1966

*Dicerura unidentata* Spungis, 1987

***DIRHIZA*** Loew, 1850

*Dirhiza lateritia* Loew, 1850

***HILVERSIDIA*** Mamaev, 1966

*Hilversidia autumnalis* Mamaev, 1966

***Paratetraneuromyia*** Spungis, 1987

*Paratetraneuromyia vernalis* Spungis, 1987

***Solntsevia*** Mamaev, 1965

*Solntsevia nigripes* Mamaev, 1965

***TETRANEUROMYIA*** Mamaev, 1964

*Tetraneuromyia lenticularis* (Spungis, 1987)

tribe Porricondylini Kieffer, 1913

= Holoneurini Enderlein, 1936

***Arctepidosis*** Mamaev, 1990

*Arctepidosis paneliusi* Mamaev & Zaitzev, 1998

***Cassidoides*** Mamaev, 1960

*Cassidoides fulvus* (Kieffer, 1896)

= *pini* Mamaev, 1960

***Claspettomyia*** Grover, 1964

= *Pachylabis* Panelius, 1965

*Claspettomyia hamata* (Felt, 1907)

*Claspettomyia niveitarsis* (Zetterstedt, 1850)

*Claspettomyia rossica* Mamaev, 1998

*Claspettomyia ussuriensis* Mamaev, 1998

***Coccopsilis*** Harris, 2004

= *Coccopsis* Meijere preocc.

*Coccopsilis marginata* (Meijere, 1901)

*Coccopsilis obscura* (Mamaev, 1964)

*Coccopsilis paneliusi* (Yukawa, 1971)

***DENDREPIDOSIS*** Mamaev, 1990

*Dendrepidosis longipennis* (Spungis, 1981)

***Divellepidosis*** Fedotova & Sidorenko, 2007

*Divellepidosis armilla* (Mamaev, 1994)

*Divellepidosis fuscostriata* (Panelius, 1965)

*Divellepidosis hypoxantha* (Panelius, 1965)

*Divellepidosis lutescens* (Spungis, 1981)

*Divellepidosis pallescens* (Panelius, 1965)

*Divellepidosis taigacola* Jaschhof, 2013

***JAMALEPIDOSIS*** Mamaev, 1990

*Jamalepidosis spungisi* Jaschhof, 2013

***Monepidosis*** Mamaev, 1966

*Monepidosis furcata* Mamaev, 1966

*Monepidosis pectinata* Mamaev, 1966

***Paneliusia*** Jaschhof, 2013

*Paneliusia albimanoides* Jaschhof, 2013

*Paneliusia aurantiaca* (Panelius, 1965)

= *modesta* (Spungis, 1981)

***Parepidosis*** Kieffer, 1913

*Parepidosis arcuata* Mamaev, 1964

= *longinodis* Panelius, 1965

*Parepidosis venustior* Gagné, 2004

***PAURODYLA*** Jaschhof, 2013

*Paurodyla tyresta* Jaschhof, 2013

***Porricondyla*** Rondani, 1840

*Porricondyla fulvescens* Panelius, 1965

*Porricondyla neglecta* Mamaev in Mamaev & Krivosheina, 1965

*Porricondyla nigripennis* (Meigen, 1830)

*Porricondyla rufescens* Panelius, 1965

***Pseudepidosis*** Mamaev, 1966

*Pseudepidosis trifida* Mamaev, 1966

***Rostellatyla*** Jaschhof, 2013

*Rostellatyla rostellata* (Panelius, 1965)

***Rostratyla*** Jaschhof, 2013

*Rostratyla globosa* (Spungis, 1981)

***Schistoneurus*** Mamaev, 1964

*Schistoneurus impressus* Mamaev, 1964

*Schistoneurus irregularis* Mamaev, 1964

***Serratyla*** Jaschhof, 2013

*Serratyla furcata* (Mamaev, 2001)

*Serratyla pubescens* (Walker, 1856)

*Serratyla spinosa* Jaschhof, 2013

***Spungisomyia*** Mamaev & Zaitzev, 1996

*Spungisomyia media* (Spungis, 1981)

***Zaitzeviola*** Fedotova & Sidorenko, 2007

*Zaitzeviola latistylata* Jaschhof, 2013

*Zaitzeviola pilosistylata* Jaschhof, 2013

*Zaitzeviola rufocinerea* (Panelius, 1965)

Cecidomyiinae Newman, 1834

supertribe Cecidomyiidi Rübsaamen & Hedicke, 1925

tribe Aphidoletini Harris, 1966

***APHIDOLETES*** Kieffer, 1904

*Aphidoletes aphidimyza* (Rondani, 1847)

*Aphidoletes urticaria* (Kieffer, 1895)

***MONOBREMIA*** Kieffer, 1912

*Monobremia subterranea* (Kieffer, 1898)

tribe Asphondyliini Rübsaamen & Hedicke, 1925

***ASPHONDYLIA*** Loew, 1850

*Asphondylia serpylli* Kieffer, 1898

= *thymi* Kieffer, 1898

***KIEFFERIA*** Mik, 1895

*Kiefferia pericarpiicola* (Bremi, 1847)

***SCHIZOMYIA*** Kieffer, 1889

*Schizomyia galiorum* Kieffer, 1889

tribe Cecidomyiini Rübsaamen & Hedicke, 1925

***ANISOSTEPHUS*** Rübsaamen, 1917

*Anisostephus betulinus* (Kieffer, 1889)

***CECIDOMYIA*** Meigen, 1803

*Cecidomyia pini* (De Geer, 1776)

***CONTARINIA*** Rondani, 1860

*Contarinia coryli* (Kaltenbach, 1859)

*Contarinia craccae* Loew, 1850

*Contarinia gei* Kieffer, 1909

= *geicola* Rübsaamen, 1917

*Contarinia kanervoi* Barnes, 1958

*Contarinia lonicerearum* (F. Löw, 1877)

*Contarinia loti* (De Geer, 1776)

*Contarinia merceri* Barnes, 1930

*Contarinia petioli* (Kieffer, 1898)

*Contarinia pisi* (Loew, 1850)

*Contarinia quercina* (Rübsaamen, 1890)

*Contarinia sambuci* (Kaltenbach, 1873)

= *lonicerearum* (F. Löw, 1874)

*Contarinia tiliarum* (Kieffer, 1890)

*Contarinia tritici* (Kirby, 1798)

*Contarinia vincetoxici* Kieffer, 1909

***MACRODIPLOSIS*** Kieffer, 1895

*Macrodiplosis pustularis* (Bremi, 1847)

= *dryobia* (Löw, 1877)

*Macrodiplosis roboris* (Hardy, 1854)

= *volvens* Kieffer, 1895

***PLEMELIELLA*** Seitner, 1908

*Plemeliella abietina* Seitner, 1908

***STENODIPLOSIS*** Reuter, 1895

*Stenodiplosis geniculati* Reuter, 1895

***THECODIPLOSIS*** Kieffer, 1895

*Thecodiplosis brachyntera* (Schwägrichen, 1835)

tribe Clinodiplosini Enderlein, 1936

***AMETRODIPLOSIS*** Rübsaamen, 1910

*Ametrodiplosis thalictricola* (Rübsaamen, 1895)

***CLINODIPLOSIS*** Kieffer, 1894

*Clinodiplosis cilicrus* (Kieffer, 1889)

***SITODIPLOSIS*** Kieffer, 1913

*Sitodiplosis mosellana* (Géhin, 1857)

tribe Hormomyiini Rübsaamen & Hedicke, 1925

***PLANETELLA*** Westwood, 1840

Planetella ?grandis (Meigen, 1804)

tribe Lestodiplosini Harris, 1966

***LESTODIPLOSIS*** Kieffer, 1894

*Lestodiplosis pallidicornis* Kieffer, 1898

unplaced Cecidomyiidi

***HARMANDIOLA*** Skuhravá, 1997

= *Harmandia* Kieffer, 1896 preocc.

*Harmandiola globuli* (Rübsaamen, 1889)

*Harmandiola tremulae* (Winnertz, 1853)

= *loewi* (Rübsaamen, 1917)

***HYGRODIPLOSIS*** Kieffer, 1912

*Hygrodiplosis vaccinii* (Kieffer, 1897)

***MASSALONGIA*** Kieffer, 1897

*Massalongia rubra* (Kieffer, 1890)

***RESSELIELLA*** Seitner, 1906

= *Thomasiniana* Strand, 1927

*Resseliella ribis* (Marikovskij, 1956)

*Resseliella theobaldi* (Barnes, 1927)

***XYLODIPLOSIS*** Kieffer, 1894

*Xylodiplosis nigritarsis* (Zetterstedt, 1850)

supertribe Lasiopteridi Rübsaamen & Hedicke, 1925

tribe Dasineurini Rübsaamen & Hedicke, 1925

***DASINEURA*** Rondani, 1840

*Dasineura affinis* (Kieffer, 1886)

*Dasineura alopecuri* (Reuter, 1895)

*Dasineura angelicae* Rübsaamen, 1916

*Dasineura cardaminis* (Winnertz, 1853)

*Dasineura engstfeldi* (Rübsaamen, 1889)

*Dasineura epilobii* (F. Löw, 1889)

*Dasineura fraxinea* Kieffer, 1907

*Dasineura fraxini* (Bremi, 1847)

*Dasineura galiicola* (F. Löw, 1880)

*Dasineura gentneri* Pritchard, 1953

*Dasineura hygrophila* (Mik, 1883)

*Dasineura hyperici* (Bremi, 1847)

*Dasineura jaapi* Rübsaamen, 1914

*Dasineura kiefferiana* (Rübsaamen, 1891)

*Dasineura leguminicola* (Lintner, 1879)

*Dasineura lotharingiae* (Kieffer, 1888)

*Dasineura mali* (Kieffer, 1904)

*Dasineura napi* (Loew, 1850)

= *brassicae* (Winnertz, 1853)

*Dasineura populeti* (Rübsaamen, 1889)

*Dasineura pteridicola* (Kieffer, 1901)

*Dasineura pteridis* (Muller, 1871)

= *filicina* (Kieffer, 1889)

*Dasineura pustulans* (Rübsaamen, 1889)

*Dasineura pyri* (Bouché, 1847)

*Dasineura ribis* Barnes, 1940

*Dasineura rosae* (Bremi, 1847)

= *Wachtliella rosarum* (Hardy, 1854)

*Dasineura similis* (F. Löw, 1888)

*Dasineura sisymbrii* (Schrank, 1803)

*Dasineura tetensi* (Rübsaamen, 1892)

*Dasineura thomasiana* (Kieffer, 1888)

*Dasineura tiliae* (Schrank, 1803)

= *tiliamvolvens* (Rübsaamen, 1889)

*Dasineura trifolii* (F. Löw, 1874)

*Dasineura ulmaria* (Bremi, 1847)

*Dasineura urticae* (Perris, 1840)

*Dasineura viciae* (Kieffer, 1888)

*Dasineura violae* (F. Löw, 1880)

***GEOCRYPTA*** Kieffer, 1913

*Geocrypta galii* (Loew, 1850)

***GEPHYRAULUS*** Rübsaamen, 1915

*Gephyraulus raphanistri* (Kieffer, 1886)

***GIRAUDIELLA*** Rübsaamen, 1915

*Giraudiella inclusa* (Frauenfeld, 1862)

***JAAPIELLA*** Rübsaamen, 1915

*Jaapiella loticola* (Rübsaamen, 1889)

*Jaapiella veronicae* (Vallot, 1827)

***KALTENBACHIOLA*** Hedicke, 1938

*Kaltenbachiola strobi* (Winnertz, 1853)

***MACROLABIS*** Kieffer, 1892

*Macrolabis cirsii* (Rübsaamen, 1890)

*Macrolabis orobi* (F. Löw, 1877)

***RABDOPHAGA*** Westwood, 1847

*Rabdophaga dubiosa* Kieffer, 1913

*Rabdophaga gemmicola* (Kieffer, 1896)

*Rabdophaga heterobia* (Loew, 1850)

*Rabdophaga iteobia* (Kieffer, 1890)

*Rabdophaga marginemtorquens* (Bremi, 1847)

*Rabdophaga nervorum* (Kieffer, 1895)

*Rabdophaga rosaria* (Loew, 1850)

*Rabdophaga salicis* (Schrank, 1803)

*Rabdophaga terminalis* (Loew, 1850)

***WACHTLIELLA*** Rübsaamen, 1915

*Wachtliella caricis* (Loew, 1850)

= *riparia* (Winnertz, 1853)

= *muricatae* (Meade, 1886)

*Wachtliella persicariae* (Linnaeus, 1767)

tribe Lasiopterini Rübsaamen & Hedicke, 1925

***LASIOPTERA*** Meigen, 1818

*Lasioptera populnea* Wachtl, 1883

*Lasioptera rubi* (Schrank, 1803)

tribe Oligotrophini Rübsaamen & Hedicke, 1925

***OLIGOTROPHUS*** Latreille, 1805

*Oligotrophus juniperinus* (Linnaeus, 1758)

*Oligotrophus panteli* Kieffer, 1898

tribe Poomyini Rübsaamen & Hedicke, 1925

***MAYETIOLA*** Kieffer, 1896

*Mayetiola avenae* (Marchal, 1895)

*Mayetiola destructor* (Say, 1817)

*Mayetiola joannisi* Kieffer, 1896

tribe Rhopalomyiini Harris, 1966

***RHOPALOMYIA*** Rübsaamen, 1892

*Rhopalomyia chrysanthemi* (Ahlberg, 1939)

*Rhopalomyia millefolii* (Loew, 1850)

*Rhopalomyia ptarmicae* (Vallot, 1849)

*Rhopalomyia tanaceticola* (Karsch, 1879)

*Rhopalomyia tubifex* (Bouché, 1847)

unplaced Lasiopteridi

***CYSTIPHORA*** Kieffer, 1892

*Cystiphora sanguinea* (Bremi, 1847)

= *hieracii* (F. Löw, 1874)

= *pilosellae* Kieffer, 1892

*Cystiphora sonchi* (Vallot, 1827)

***ITEOMYIA*** Kieffer, 1913

*Iteomyia capreae* (Winnertz, 1853)

***SEMUDOBIA*** Kieffer, 1913

*Semudobia betulae* (Winnertz, 1853)

## Excluded species

*Anarete lacteipennis* Kieffer, 1906. Misidentification.

*Camptomyia antennata* Felt, 1920. Misidentification.

*Camptomyia maxima* Mamaev, 1961. Presumptive misidentification; Finnish record cannot be verified.

*Camptomyia piceae* Panelius, 1965. Presumptive misidentification; Finnish record cannot be verified.

*Catarete brevinervis* (Zetterstedt, 1851). Misidentification.

*Cecidomyia aurora* Mannerheim, 1823. Nomen dubium.

*Epidosis flavescens* F. Löw, 1874. Nomen dubium.

## Note

***Anarete* sp. 2**. This species was described by Jaschhof and Jaschhof (2009) but not named. Identification of *Anarete* species is practically impossible using the keys and descriptions available in the literature.
